# Transmission of viable *Haemophilus ducreyi* by *Musca domestica*

**DOI:** 10.1371/journal.pntd.0012194

**Published:** 2024-05-30

**Authors:** Haley D. Stabile, Kayla McCandless, Rachel A. Donlan, Jordan R. Gaston, Tricia L. Humphreys

**Affiliations:** 1 Department of Biology, Allegheny College, Meadville, Pennsylvania, United States of America; 2 Lake Erie College of Osteopathic Medicine, Erie, Pennsylvania, United States of America; 3 Krystal Biotech, Inc., Pittsburgh, Pennsylvania, United States of America; 4 University of Pittsburgh Medical Center, Department of Microbiology and Molecular Genetics, Pittsburgh, Pennsylvania, United States of America; 5 University of Pittsburgh Medical Center, St. Margaret Hospital, Pittsburgh, Pennsylvania, United States of America; Yale University School of Medicine, UNITED STATES

## Abstract

*Haemophilus ducreyi* was historically known as the causative agent of chancroid, a sexually-transmitted disease causing painful genital ulcers endemic in many low/middle-income nations. In recent years the species has been implicated as the causative agent of nongenital cutaneous ulcers affecting children of the South Pacific Islands and West African countries. Much is still unknown about the mechanism of *H*. *ducreyi* transmission in these areas, and recent studies have identified local insect species, namely flies, as potential transmission vectors. *H*. *ducreyi* DNA has been detected on the surface and in homogenates of fly species sampled from Lihir Island, Papua New Guinea. The current study develops a model system using *Musca domestica*, the common house fly, as a model organism to demonstrate proof of concept that flies are a potential vector for the transmission of viable *H*. *ducreyi*. Utilizing a green fluorescent protein (GFP)-tagged strain of *H*. *ducreyi* and three separate exposure methods, we detected the transmission of viable *H*. *ducreyi* by 86.11% ± 22.53% of flies sampled. Additionally, the duration of *H*. *ducreyi* viability was found to be directly related to the bacterial concentration, and transmission of *H*. *ducreyi* was largely undetectable within one hour of initial exposure. Push testing, Gram staining, and PCR were used to confirm the identity and presence of GFP colonies as *H*. *ducreyi*. This study confirms that flies are capable of mechanically transmitting viable *H*. *ducreyi*, illuminating the importance of investigating insects as vectors of cutaneous ulcerative diseases.

## Author summary

Cutaneous ulcers are prevalent throughout the world, especially in low/middle-income countries. The commonality of diseases that cause cutaneous ulcers makes the transmission mechanisms of these diseases of great interest to public health. Understanding the mechanism by which these diseases are contracted and transmitted greatly informs prevention and treatment strategies. This study investigates the potential for mechanical transmission of a bacterial pathogen, *Haemophilus ducreyi*, using the common house fly, *Musca domestica*. Mechanical transmission via insects is the process by which a pathogen adheres to the appendages or mouthparts of a vector, thereby allowing the vector to spread the pathogen from infected to uninfected individuals. During this study, live *H*. *ducreyi* was found to transfer from the surface of *M*. *domestica* onto agar plates via the flies’ innate behavior alone, suggesting flies are capable of transmitting the bacteria. This novel finding for *H*. *ducreyi* should be carefully considered in future prevention and treatment strategies for cutaneous ulcers caused by this pathogen and serves as a model for similar diseases.

## Introduction

*Haemophilus ducreyi* is a gram-negative coccobacillus that was first identified as the causative agent of chancroid in 1889 [1]. Chancroid is a sexually transmitted disease that presents as painful genital ulcers in which inflammatory papules form and cause crater-like ulcers upon pustule rupture [2]. If left untreated, the open wounds increase the individual’s risk of contracting other bacterial and viral infections, such as human immunodeficiency virus [3]. Chancroid was highly prevalent in many low/middle-income countries in the 1980s and 1990s, with smaller spillover outbreaks occurring in high-income countries such as the United States in that era [2]. Since the 2000s, the global incidence of chancroid has declined, albeit whether this is secondary to a true decline in cases or decrease in surveillance is unclear [4].

More recently, *H*. *ducreyi* has been identified as the causative agent of skin ulcers primarily affecting the lower limbs of children [5] within the yaws-endemic regions of Papua New Guinea, Ghana, the Solomon Islands, and Vanuatu [4]. Instances of nongenital cutaneous ulcers caused by *H*. *ducreyi* have also been documented in industrialized countries as a result of tourism and globalization [6]. This new mode of *H*. *ducreyi* infection was first documented in 1989, when a 22-year old male from Denmark presented with a pedal ulcer after returning from the Fiji Islands [7]. Extragenital sites of *H*. *ducreyi* infection are routinely studied in the human challenge model (established in 1994) in which subjects are inoculated on the upper arm [8]. Cutaneous ulcers can be transmitted through direct contact of an infected ulcer with breaks in the skin barrier or mucous membranes, leaving children in regions who wear fewer layers of clothing secondary to the tropical climates and who have minimal access to health services at high risk for infection [9]. The recognition of *H*. *ducreyi* as a causative agent of skin ulcerations is increasing in yaws endemic regions; *H*. *ducreyi* was estimated to affect 2% of the entire population and greater than 7% of 5–15 year old children in Papua New Guinea [5]. A more recent study sampling children of Papua New Guinea detected *H*. *ducreyi* in approximately 97% of ulcerations less than 1 cm in diameter within the sampled population, suggesting that *H*. *ducreyi* is a primary rather than secondary cause of infection [10].

Originally, these skin ulcerations were thought to be a reemergence of yaws [5], an infection of the dermis and bone caused by *Treponema pallidum* subsp. *pertenue* [11]. *H*. *ducreyi-*infected lesions mimic some of the clinical features of yaws lesions, making differentiation and diagnosis require PCR-based testing and/or culture [12]. In 2012, the World Health Organization (WHO) launched a resolution for the eradication of yaws by 2020. The strategy for eradication consisted of mass treatment of endemic areas with azithromycin. However, these efforts have been complicated by the presence of *H*. *ducreyi* in cutaneous ulcers, which requires laboratory tests to confirm [5]. Additionally, there is a report of azithromycin-resistant *T*. *pallidum* subsp. *pertenue*, which could hinder future efforts if resistance spreads [13]. Although a single dose of azithromycin should be effective in the treatment of *H*. *ducreyi* ulcers [14], these infections have nevertheless persisted, suggesting that an environmental reservoir may harbor these pathogens.

While there is no documented environmental reservoir of *H*. *ducreyi*, flies have been implicated as a possible mode of transmission. Local insects have been described qualitatively associating with infected ulcers in addition to *H*. *ducreyi* DNA being detected on flies via PCR analysis [15,5]. Flies have been confirmed as vectors of other bacteria through mechanical transmission [16,17,18], including the transmission of yaws, though the evidence for transmission of yaws is limited to experimental models [19].

Mechanical transmission of a pathogen is a process by which species from an infected host adhere to the appendages or mouthparts of a vector and are then transmitted to a new host [20]. Due to the similarities in pathogenesis, population, and locale between cutaneous ulcers caused by *T*. *pallidum* subsp. *pertenue* and *H*. *ducreyi*, the documented transmission of yaws by flies, and recent data detecting *H*. *ducreyi* DNA on insect species in cutaneous ulcer endemic areas, implication of flies as a possible vector of *H*. *ducreyi* is reasonable. This implication is further supported by the finding of *H*. *ducreyi* DNA on asymptomatic individuals, bed linens, and flies found in proximity to infected individuals [15]. The presence of DNA is compelling, but does not confirm cell viability. Therefore, without culturing bacteria from the flies, flies could not be confirmed as a transmission vector or an environmental reservoir of *H*. *ducreyi* [21].

*Musca domestica*, the common house fly, has been reported to transmit *T*. *pallidum* subsp. *pertenue* from infected to uninfected individuals [21]. This fly species is used as a model system for studying the mechanical transmission of *Bacillus anthracis*, a cutaneous pathogen that causes the ulcerative disease anthrax in humans and cattle [22], as well as other bacterial pathogens, such as *Salmonella*, *Staphylococcus aureus*, and *Escherichia coli* [21]. The infectious potential of *M*. *domestica* can be attributed to its regurgitation and defecation behaviors following the ingestion of pathogens and to the hairy structures and sticky pads of its feet [23].

Here we demonstrate *M*. *domestica* as a possible vector of *H*. *ducreyi* by detecting the transmission of live *H*. *ducreyi* from flies onto chocolate agar plates. Having a deeper understanding of the transmission mechanisms of *H*. *ducreyi* could inform prevention and treatment strategies for cutaneous ulcers caused by *H*. *ducreyi* as well as similar cutaneous skin infections such as yaws.

## Methods

### *M*. *domestica* rearing

The first generations of *M*. *domestica* were purchased from Carolina Biological Supply Company and from Mantid Kingdom. Following generations were reared 2–3 weeks after the eclosure of the prior generation. During rearing, chicken liver was placed in a fly house to encourage adult flies to lay eggs, and an OliveTech Aroma Diffuser (400 mL) was run for approximately 6 hours daily. Flies were maintained in the fly houses at 22–25°C under natural lighting conditions and were fed a 1:1 mixture of sucrose and instant nonfat dry milk powder. Both the sugar-milk mixture and water-soaked cotton were readily available.

### *H*. *ducreyi* culture

*H*. *ducreyi* 35000HP/pRB157K expresses green fluorescent protein (GFP) and streptomycin resistance [24]. Bacteria were cultured on enriched chocolate agar plates (GC agar base supplemented with 1% (w/v) hemoglobin, 68.43 nM L-glutamine, 147.47 nM L-cysteine-HCL monohydrate, 1% (w/v) dextrose, and 100 μg/mL streptomycin). *H*. *ducreyi* colonies were detected by shining a Vansky 51 LED UV Flashlight on exposed plates. The bacterial stock was stored at -80°C, and bacteria were revived prior to each trial on enriched chocolate agar incubated at 35°C with 5% CO_2_.

### Preparation of *H*. *ducreyi* for exposure

Approximately 15 minutes prior to *M*. *domestica* exposure, *H*. *ducreyi* was suspended in 0.85% (w/v) NaCl. The turbidity of the bacterial suspension was approximated using a 0.5 McFarland Standard. The target concentration of the suspension was 2.3 x 10^5^ CFU/mL, since the approximate concentration of *H*. *ducreyi* detected in human pustules is 1.8 x 10^5^–3.6 x 10^5^ CFU [25]. It is challenging to make precise suspensions of *H*. *ducreyi* because the cells clump. Serial dilutions were performed to find the actual concentration of the suspension for each exposure.

Control and experimental vials (Pyrex, 40 mL) were prepared by placing half of a large cotton ball (Fisherbrand, non-sterile) at the bottom of each vial, and a full cotton ball in the top of the vial. Vials were sterilized prior to use. Bottom cotton balls of control vials were saturated with a sterile 2.5% (w/v) sugar-milk-water (SMW) mixture, while bottom cotton balls of experimental vials were saturated with a 1:1 SMW / bacterial suspension solution. Top cotton balls were dipped in the same solutions in 60 mm Petri dishes.

### *M*. *domestica* exposure

Prior to *H*. *ducreyi* exposure, flies were removed from the fly house using a Sokos Humane Insects and Bug Catcher and transferred into sterile vials. Experimental flies and control flies were contained separately. Flies were fasted for 4 hours to encourage subsequent feeding on the suspension. After the fasting period, flies were anesthetized with gaseous CO_2_ for 10–20 seconds (until no movement was observed) to ensure easy handling from vial to vial. Flies were then transferred into the appropriate vial (control or experimental), and permitted to feed on the solutions for 30 minutes. Following the exposure period, flies were again anesthetized and transferred onto separate enriched chocolate agar plates for 10 minutes. Flies were permitted to walk freely on the plates, but were freed by gentle pushing to an upright position if they were immobilized by sticking to the agar. All fly handling was performed using BioQuip soft forceps sterilized in 10% (v/v) bleach. After each trial, gaseous CO_2_ was injected into each plate to anesthetize flies for easy transfer to the fly morgue (a container filled with 70% (v/v) ethanol) in order to safely dispose of flies without risk of contamination. Enriched chocolate agar plates were incubated at 35° C in a 5% CO_2_ incubator for 3–5 days.

### Exposure methods

The transmission of *H*. *ducreyi* by *M*. *domestica* was investigated using three different exposure methods: an Individual Exposure method in which a separate vial was utilized to expose each fly, a Group Exposure method in which 5 flies were placed into a single vial for exposure, and a Timed Trials method in which 5 flies were placed into a single vial for exposure, but the flies were transferred to subsequent plates following the initial plating (S1 Fig). Within Individual Exposure trials, 5 experimental flies and 5 control flies were individually exposed to the appropriate solution, totaling 10 vials per trial (n = 40 over 4 trials). Group Exposure trials were conducted by preparing 2 exposure vials and 2 control vials, containing 5 flies each (n = 60 over 3 trials). Since flies are not individually isolated in their natural environment, they were not individually isolated during the group exposures. As in the Group Exposure trials, experimental flies and control flies (n = 110 over 11 trials) were exposed to the appropriate suspension in separate vials each containing 5 flies during the Timed Trials. However, instead of experimental flies being directly transferred to the fly morgue after the initial 10 minute plating period, each of the flies were then transferred to a new enriched chocolate agar plate every 10 minutes. This process was repeated 4 times for a total of 5 time points. Initially, the time points progressed from 0 minutes (the initial plating) to 120 minutes, in which the flies were transferred into separate, sterile vials for 20 minutes between platings. However, after a few trials it became apparent that the viability of *H*. *ducreyi* was primarily limited to 60 minutes after initial exposure. Therefore, the final time point was reduced to 40 minutes.

### Identification of green colonies as *H*. *ducreyi*

Following exposure to live *H*. *ducreyi*, flies were placed on agar plates and permitted to walk freely, mechanically transmitting bacteria to the agar surface. Plates were considered positive for the presence of *H*. *ducreyi* if they exhibited at least 1 GFP colony. Control plates of all trials were negative, as they did not exhibit any GFP colonies. Sample sizes reflect the number of experimental flies utilized within the exposure method, and for the Timed Trials, all experimental plates corresponding to the same experimental fly were recorded as one data point for combined averages; if at least one experimental plate through all time points corresponding to the same experimental fly was positive for the presence of *H*. *ducreyi*, then all experimental plates corresponding to that fly were recorded once as positive for the presence of *H*. *ducreyi*.

### Push test and genomic DNA extraction

**Fig 1:**
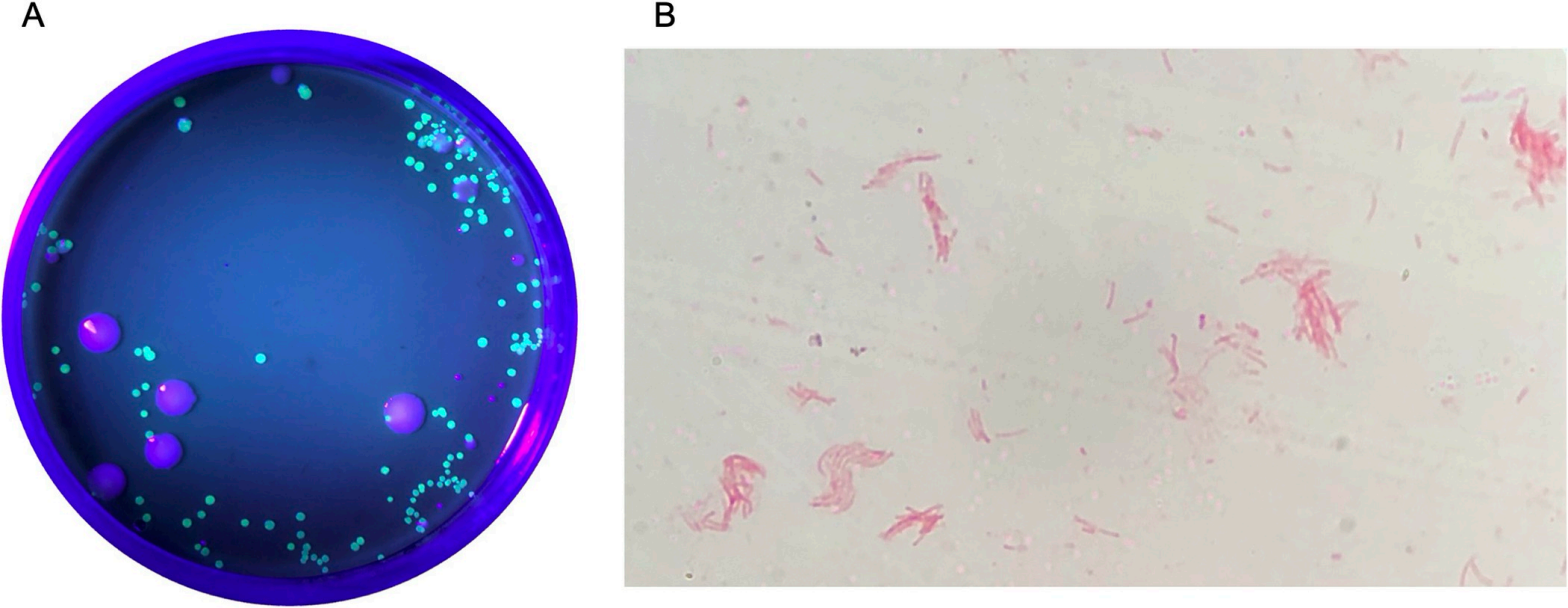
Identification of *H*. *ducreyi*. (A) Visualization of an experimental plate under UV light. *H*. *ducreyi* colonies appear green due to the expression of GFP, while other microbiota appear purple. (B) Visualization of *H*. *ducreyi* following Gram staining. Gram staining allowed for confirmation of the sample as *H*. *ducreyi*, as the cells appear as pink coccobacilli arranged in parallel rows. The patterns depicted are often referred to as “school-of-fish” or “fingerprint” patterns [27].

After 72 hours of incubation, 2 GFP colonies from each experimental plate (Fig 1A) were chosen for push testing. Select non-GFP colonies were extracted from control plates as well. Colonies were pushed using a flat-edge toothpick, and passed the push test if they could be pushed intact across the surface of the medium [26]. Colonies were then extracted from the medium and transferred to a PCR tube containing nuclease-free (NF) water. All samples were heated to 98°C for 5 minutes in a BioRad MyCycler to extract genomic DNA. Samples were stored at -20°C until PCR was performed.

### Gram staining

Gram staining was another technique employed to confirm the identity of *H*. *ducreyi*. One colony was tested from 2 experimental and 2 control plates from the Group Exposure trials (n = 12 over 3 trials), while one colony from an experimental plate and a control plate were tested from the Individual Exposure trials (n = 8 over 4 trials). Gram staining was not performed for the Timed Trials. Pure cultures of *H*. *ducreyi* were utilized as positive controls, while *Corynebacterium hoffmanni*, *Staphylococcus citreus*, *Staphylococcus epidermidis*, and *Serratia marcescens* were used as staining controls. *H*. *ducreyi* was identified by morphological and physiological characteristics under a microscope, including the observation of pink gram-negative rods arranged in parallel rows (Fig 1B), referred to as “school-of-fish” or “fingerprint” patterns [27].

### PCR, gel electrophoresis, and sequencing

The *pal* gene, which is unique to *H*. *ducreyi* [28], was amplified by PCR following genomic DNA extraction from samples. The master mix utilized for each PCR reaction included the following reagents: 2x New England BioLabs Inc. OneTaq DNA Polymerase, forward primer (10 μM, 5’-AGTAGTTCATCAGGTAAAACAGATG-3’ [29], reverse primer (10 μM, 5’-AAATTAGTACTCTAATACTGCACGG-3’ [29], and NF water. *H*. *ducreyi* DNA was used as a positive control, while a sample with no template was used as a negative control. PCR was performed in a BioRad MyCycler under the following conditions: 94°C for 30 seconds (1 cycle), 94°C for 30 seconds, 55°C for 30 seconds, 68°C for 1 minute (40 cycles), 68°C for 5 minutes (1 cycle), held at 4°C. Gel electrophoresis was performed using 1% (w/v) agarose to ensure the presence or absence of the *pal* band at 423 bp. Sequencing of selected samples (n = 6) was performed by Eurofins Genomics, LLC following purification using ThermoFisher ExoSAP-IT according to manufacturer’s directions.

## Results

**Fig 2:**
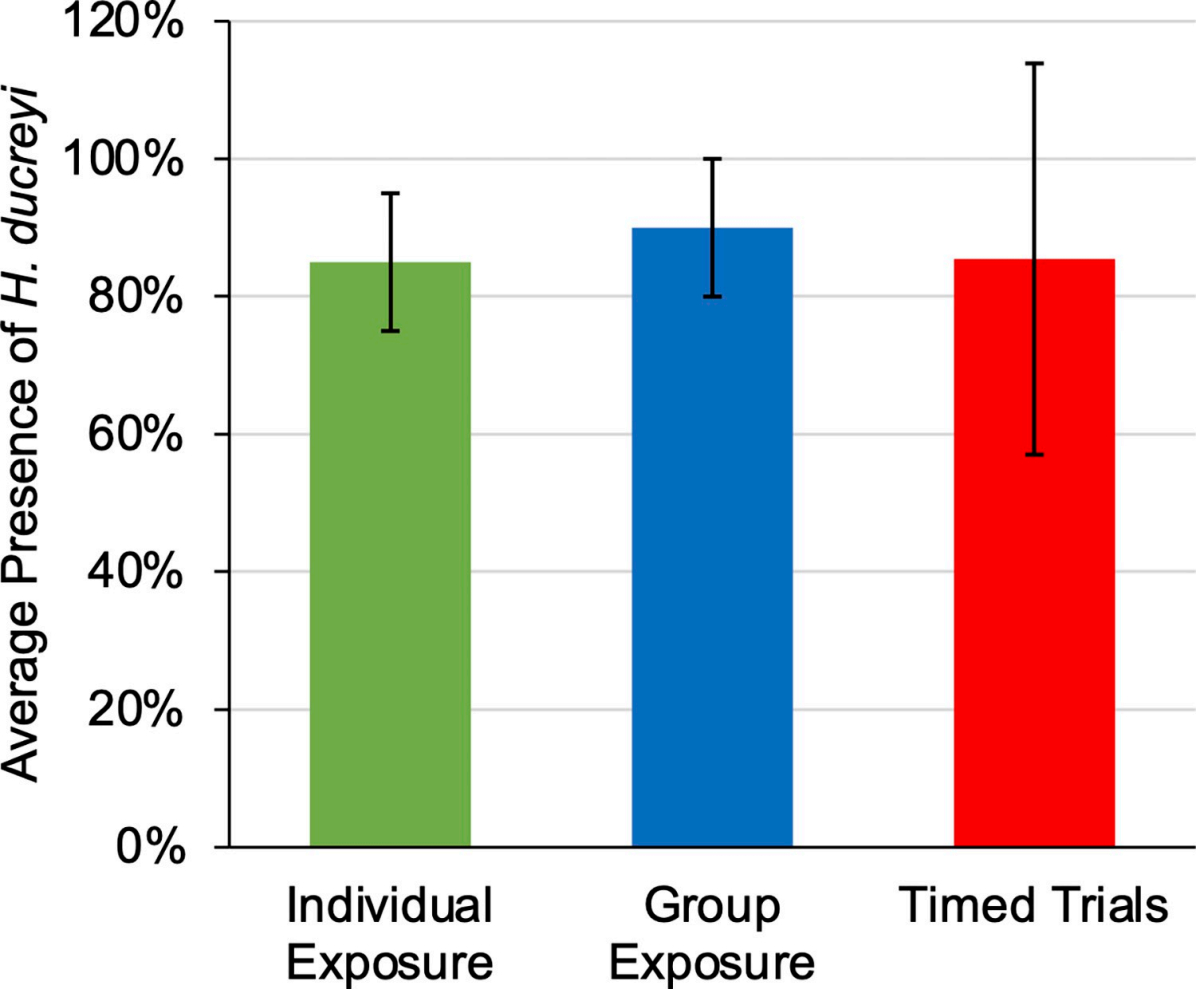
The presence of *H*. *ducreyi*-GFP colonies (mean ± SD) across exposure methods. Plates with at least 1 GFP colony were included as positive for the presence of *H*. *ducreyi*. Control plates are not included in these data, as they did not exhibit any GFP colonies. The number of exposed flies and the number of trials conducted were as follows: n = 20 over 4 trials (Individual Exposure), n = 30 over 3 trials (Group Exposure), n = 55 over 11 trials (Timed Trials).

The transmission of *H*. *ducreyi* by *M*. *domestica* was investigated using three different exposure methods: Individual Exposure, Group Exposure, and Timed Trials. The average percentage of experimental plates positive for the presence of *H*. *ducreyi* was 86.11% ± 22.53%. Considering each exposure method separately, the average percentage of experimental plates positive for the presence of *H*. *ducreyi* was 85% ± 10%, 90% ± 10%, and 85.5% ± 28.4% for the Individual Exposure, Group Exposure, and Timed Trials, respectively (Fig 2 and S1 Data).

**Fig 3:**
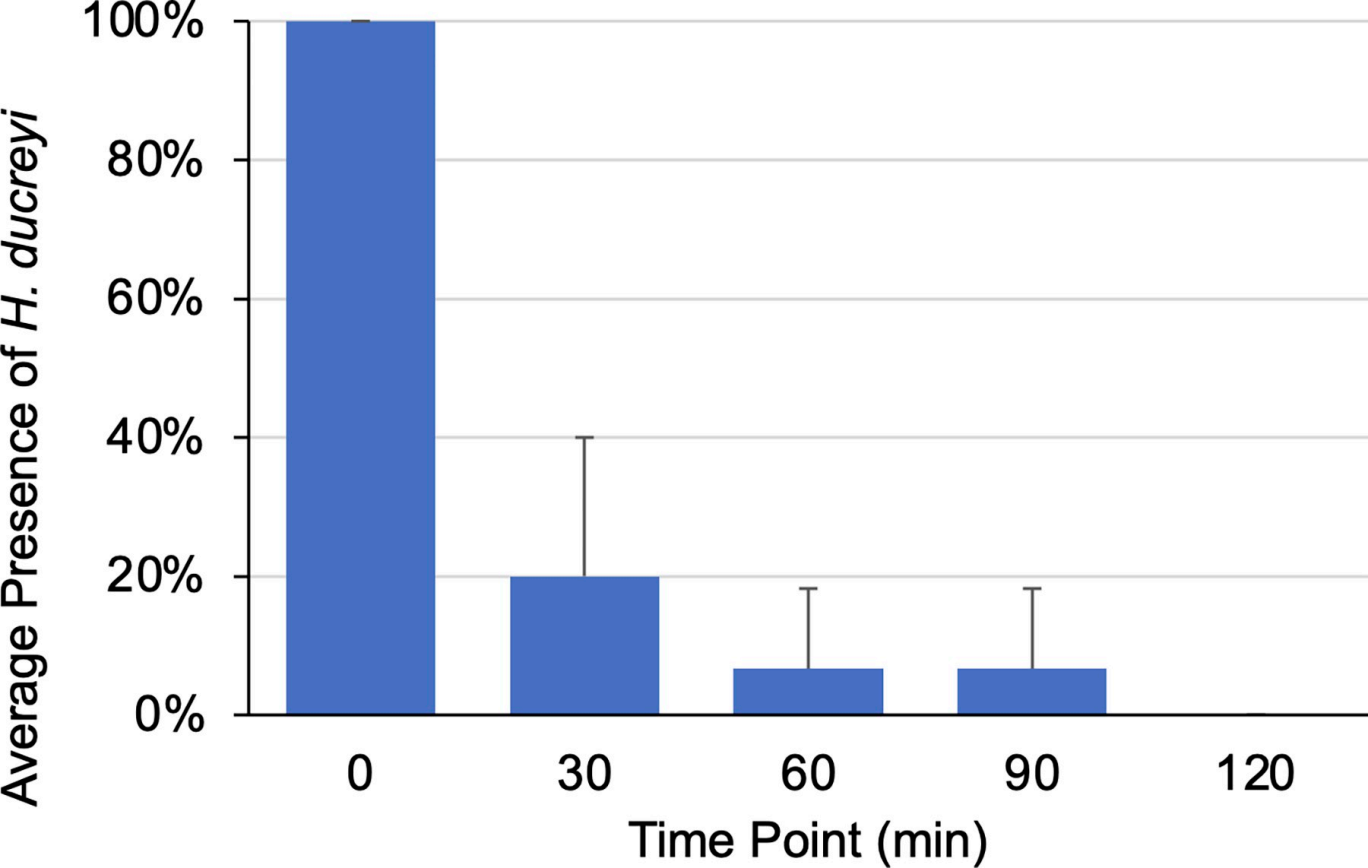
Detection of *H*. *ducreyi* (mean ± SD) at progressive time points within the Timed Trials. Control plates are not included in the data, as they did not exhibit any GFP colonies. (n = 15 over 3 trials).

**Fig 4:**
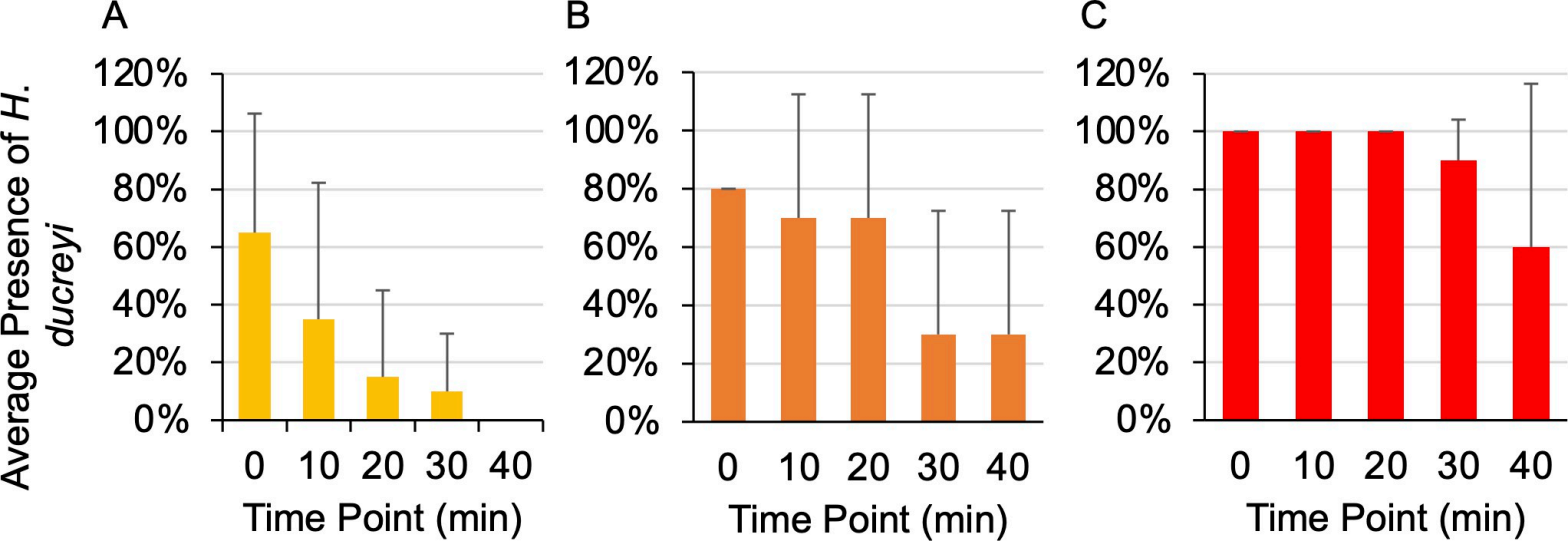
**Detection of *H*. *ducreyi*-GFP colonies (mean ± SD) within the Timed Trials**. Control plates are not included in these data, as they did not exhibit any GFP colonies. (A) Data in which the actual bacterial concentration fell below the target concentration of 2.3 x 10^5^ CFU/mL (n = 20). (B) Data in which the actual bacterial concentration fell near the target concentration in a range between 1.6 x 10^5^ to 1.5 x 10^6^ CFU/mL (n = 10). (C) Data in which the actual bacterial concentration fell well above the target concentration (n = 10).

Through the initial Timed Trials experiments, the presence of GFP colonies dramatically decreased between the initial plating and the 30 minute time point (Fig 3). Therefore, the time points were shortened to gain a more precise duration of *H*. *ducreyi* viability after transmission by *M*. *domestica*. After this adjustment, the average number of experimental plates with at least one GFP colony decreased below 50% by the 30 minute time point. The Timed Trials data suggest that the maximum duration of *H*. *ducreyi* viability following initial fly exposure is between 90 and 120 minutes (Fig 3). Because it is difficult to make precise suspensions of *H*. *ducreyi* due to clumping, the data were further analyzed by categorizing into trials that fell below the target *H*. *ducreyi* concentration (2.3 x 10^5^ CFU/mL), near the target concentration, and well above the target concentration (Fig 4). Not surprisingly, the viability duration of *H*. *ducreyi* after transmission is dose dependent, as lower bacterial concentrations result in a lower transmission of *H*. *ducreyi* at later time points while higher bacterial concentrations result in a higher transmission of *H*. *ducreyi* at the same time points.

Data collected to confirm the identity of *H*. *ducreyi* indicate that the GFP colonies are *H*. *ducreyi* colonies, while the control colonies are not. Push testing and Gram stains confirmed the presence of *H*. *ducreyi* in all tested experimental samples, and confirmed that the tested control colonies were not *H*. *ducreyi*. Additionally, PCR provided further confirmation that the GFP-expressing colonies were *H*. *ducreyi* in the majority of tested experimental samples, as 99.05% (n = 105) of experimental samples exhibited a 423 bp band. While one GFP colony did not exhibit the expected 423 bp band after PCR, the colony did pass the push test, and it is possible that the PCR reaction was inhibited by trace amounts of hemoglobin from the chocolate agar on which the colony was grown [30]. The PCR may have also been inhibited if the sample was contaminated with other microbiota. All plates, including control plates, exhibited non-GFP microbiota that could have surrounded and been collected with the GFP colony (Fig 1A), potentially inhibiting the PCR if the template was impure. Purified PCR samples sent for sequencing further identified the GFP colonies as *H*. *ducreyi*. One sample resulted in a 99.73% identity match in comparison to *pal*, while all other sequencing samples resulted in a 100% identity match. In addition, the majority of tests performed on colonies from control plates suggest that *H*. *ducreyi* was absent from these samples as 97.37% (n = 38) did not exhibit a 423 bp band. Although one control sample did test positive for the presence of *pal* through PCR, the corresponding control plate did not exhibit any GFP colonies and the collected sample did not pass the push test. Therefore, the most likely explanation for this finding is that the control sample was contaminated with *H*. *ducreyi* DNA either during collection or through the PCR process.

## Discussion

This study confirms that *M*. *domestica* are capable of mechanically transmitting viable *H*. *ducreyi*. Considering all exposure methods, the average percentage of experimental plates positive for the presence of *H*. *ducreyi* following transmission by experimental flies was 86.11% ± 22.53%. The Timed Trials exhibited a greater standard deviation than the other exposure methods (Fig 2). This finding could be a result of the varying bacterial concentrations used within the Timed Trials in comparison to the other exposure methods; while the target bacterial concentration was 2.3 x 10^5^ CFU/mL, *H*. *ducreyi* cells often clump together in liquid suspensions [31], making the approximation of bacterial concentration difficult. The Timed Trials bacterial concentrations varied from below the target concentration to well above the target concentration, potentially explaining the observed difference in standard deviation in comparison to the Group or Individual Exposures.

Large standard deviations were also observed when the Timed Trials data were categorized based on bacterial concentration (Fig 4), which could be due to the limited number of flies within each category (below n = 20, near n = 10, above n = 10). However, the sample size of experimental flies within each category is comparable to the number of examined flies within conceptually similar studies [17,15]. Even when considering the standard deviations of the data, it appears that the viability duration of *H*. *ducreyi* after transmission is dose dependent, as *H*. *ducreyi* was observed at longer time points when experimental flies were exposed to a higher bacterial concentration. In comparing the Individual Exposure and the Group Exposure methods, the data sets do not exhibit a notable difference, potentially indicating that fly-to-fly transmission of *H*. *ducreyi* is limited (Fig 2).

Detection of viable *H*. *ducreyi* is reduced quickly with only 20% of flies transferring the bacteria by 30 minutes and none by 120 minutes (Fig 3). There could be several reasons for this, including loss of viability of *H*. *ducreyi* outside its preferred growth conditions or bacteria mechanically dropping off the fly over time. *H*. *ducreyi* is a fastidious bacterium that can be challenging to grow under laboratory conditions, including a requirement for microaerophilic conditions which may be lacking on the surface of a fly. That said, these experiments were carried out under indoor laboratory conditions with controlled heating, cooling, and humidity. Tropical climates with higher humidity and warmer temperatures may allow for longer viability and a wider possible window for transmission. However, if viability in the field drops off as quickly as it does in the lab, this could limit the flies’ ability to transmit over large distances, leading to localized pockets of infection rather than widespread infection.

*H*. *ducreyi* DNA has been detected on unbroken skin, flies, and bed linens [15]. It is possible the source of the DNA is nonviable bacteria, perhaps deposited onto these surfaces by flies. If so, these surfaces would not serve as a reservoir for transmissible *H*. *ducreyi*. Cultures would need to be performed on these samples in order to distinguish between viable and nonviable bacteria but this is difficult to accomplish in remote locations with a fastidious bacterium.

Potential limitations of this study include the variance in bacterial exposure concentrations and the experimental setting in comparison to real-world conditions. However, in considering trials that were below or near the target bacterial concentration, the transmission of viable *H*. *ducreyi* by *M*. *domestica* is still apparent. It has been suggested that as few as 1 to 2 CFU are required for *H*. *ducreyi*-associated papule formation [32], and at least 1 CFU of *H*. *ducreyi* was transmitted by the majority of experimental flies exposed to a bacterial concentration below the target concentration (Fig 4A). Additionally, this study does not confirm that house flies are capable of transmitting viable *H*. *ducreyi* to human hosts. Further studies should be conducted within endemic regions to support these data, in which flies in proximity to infected individuals are cultured for the presence of live *H*. *ducreyi*.

Since house flies are found globally [33] and have previously been established as vectors of cutaneous pathogens [22,21], this study is relevant to the prevention and treatment of cutaneous ulcers resulting from *H*. *ducreyi* infection. This study provides the first documentation of *M*. *domestica* as a vector for the transmission of viable *H*. *ducreyi*, which should be considered in future efforts to control cutaneous ulcers caused by *H*. *ducreyi*.

## Supporting information

S1 Fig Flies were exposed in 3 different ways. (A) Individual Exposure, flies were exposed one at a time to *H*. *ducreyi*; n = 40. (B) Group Exposure, flies were exposed to *H*. *ducreyi* in groups of 5; n = 60. (C) Timed Trials, flies were exposed in groups of 5 as in B but additional transfers were performed to determine the length of time detection of *H*. *ducreyi* was possible; n = 110. See Methods for more details.
(PDF)

S1 Data Each tab contains the percent of flies with detectable *H*. *ducreyi* with each exposure method. Means and standard deviations are noted.
(XLSX)
