# Supplementary material for: Transmission of viable Haemophilus ducreyi by Musca domestica
Source: PLoS Negl Trop Dis. 2024 May 30;18(5):e0012194. doi: 10.1371/journal.pntd.0012194 (PMC11139276; doi:10.1371/journal.pntd.0012194)
Supplement: S1 Fig — (A) Individual Exposure, flies were exposed one at a time to H. ducreyi; n = 40. (B) Group Exposure, flies were exposed to H. ducreyi in groups of 5; n = 60. (C) Timed Trials, flies were exposed in groups of 5 as in B but additional transfers were performed to determine the length of time detection of H. ducreyi was possible; n = 110. See Methods for more details. (PDF) [file pntd.0012194.s001.pdf]

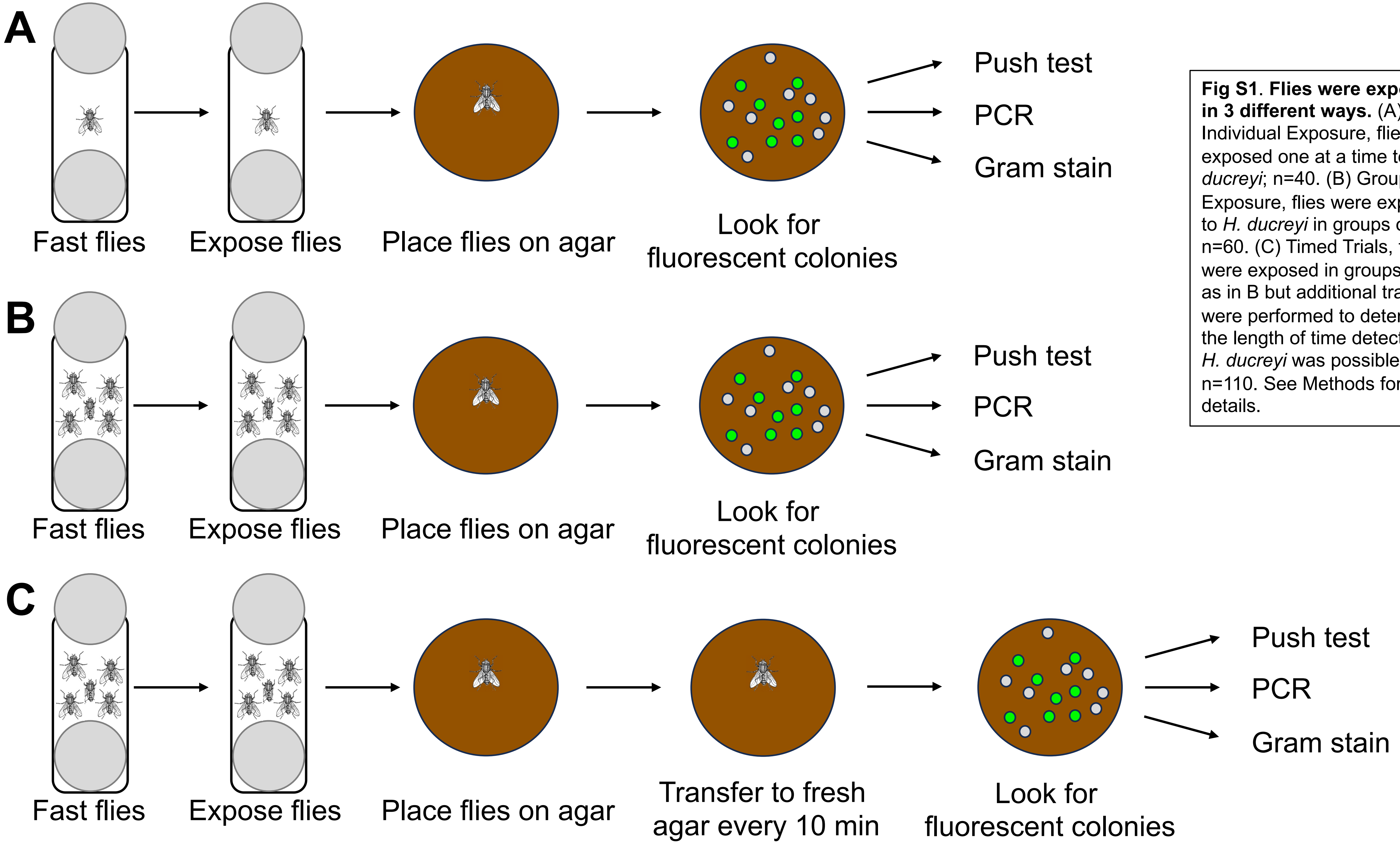

Fig S1. Flies were exposed in 3 different ways. (A) Individual Exposure, flies were exposed one at a time to *H. ducreyi*; n=40. (B) Group Exposure, flies were exposed to *H. ducreyi* in groups of 5; n=60. (C) Timed Trials, flies were exposed in groups of 5 as in B but additional transfers were performed to determine the length of time detection of *H. ducreyi* was possible; n=110. See Methods for more details.
